# Oral Sex following Abortion: Case Report of a Sexually Transmitted Infection of Group A Streptococcus Causing Peritonitis

**DOI:** 10.1155/2022/1362255

**Published:** 2022-03-31

**Authors:** Kenneth L. Chan, Louis-Patrick Haraoui, Walter Demczuk, Marc-Christian Domingo, Eric Bergeron

**Affiliations:** ^1^Department of Gynecology, Charles-LeMoyne Hospital, Greenfield Park, Canada; ^2^Department of Microbiology, Université de Sherbrooke, Canada; ^3^National Microbiology Laboratory, Public Health Agency of Canada, Winnipeg, Manitoba, Canada; ^4^Institut National de Santé Publique du Québec, Laboratoire de Santé Publique du Québec, Canada; ^5^Department of Surgery, Charles-LeMoyne Hospital, Greenfield Park, Canada

## Abstract

Group A *Streptococcus* (GAS) is a rare cause of peritonitis with only a few reports of disease associated with surgical abortion, vaginal delivery, or intrauterine devices, most of which are speculated to be in association with the female genital tract. Only a single case of GAS infection transmission through contemporary oral sex has been previously reported. We report a strange case of GAS peritonitis occurring after abortion and oral sex.

## 1. Introduction

Peritonitis primarily occurs in the absence of an abdominal source [1–4] and represents less than 1% of all peritonitis in adults [5, 6]. *Streptococcus pyogenes* (Group A *Streptococcus;* GAS) disease is unique to humans and can range from mild infections of pharyngitis and scarlet fever, to severe invasive manifestations such as bacteremia, cellulitis, necrotizing fasciitis, streptococcal toxic shock syndrome, septic arthritis, puerperal sepsis, meningitis, osteomyelitis, and endocarditis [1, 2, 7–10]. However, it is an unusual pathogen involved in peritonitis [11]. Infections due to this pathogen are increasing in incidence and severity [12]. A related mortality up to 45% has been reported [13].

Ascending inoculation from the female genital tract has been postulated as a mechanism for primary GAS peritonitis [2, 6, 7, 10]. Reported women to men ratios between 4 : 1 and 7 : 1 support the female genitalia as a probable route of infection [7, 14]. Furthermore, the prevailing incidence in women of childbearing age compared to postmenopausal women also supports the ascent of GAS from the genital tract [2, 7, 14].

There is now more evidence that GAS peritonitis, although very rare, is associated with vaginal delivery [1, 4, 9, 15], gynecologic interventions [1, 11], and intrauterine contraceptive devices (IUCD) [1, 4, 16]. Orogenital contact is a well-known means of transmission of disease but only one case of transmission has been previously reported through oral sex [16]. We hereby present another possible case of orogenital transmission of GAS in a patient who participated in oral sex with a GAS carrier sexual partner the same day after undergoing an abortion procedure and thereafter developed peritonitis.

## 2. Case Presentation

**Figure 1:**
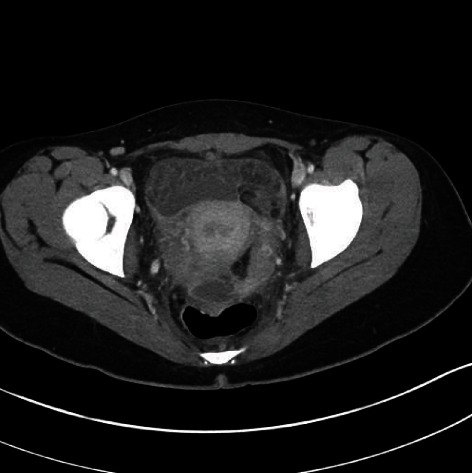
Initial enhanced abdominal CT scan showing free pelvic fluid.

A 16-year-old female without relevant medical or gynecologic history underwent a planned surgical abortion at 8 weeks of gestation. The procedure was carried out without any difficulty or evident complication. The patient presented two weeks later with severe lower abdominal pain lasting for 2 days. She was well oriented and was not hypotensive. Pulse rate was 96 and temperature 36.7°C. There was abdominal tenderness with positive rebound in the lower part of the abdomen. White cell count was 30,100/mm^3^, and CRP was 350 mg/mL (normal: 0.0-5.0 mg/mL). Blood cultures were not drawn. A CT scan showed the presence of free fluid without demonstration of a cause of peritonitis (Figure 1).

The patient was started on cefoxitin and doxycycline for a presumed diagnosis of pelvic inflammatory disease. A laparoscopy was promptly scheduled to rule out a possible uterine perforation. During the intervention, there was pus within the peritoneal cavity, mainly localized in the pelvis. There was no uterine perforation, and fallopian tubes showed inflammation but no frank evidence of abscess or infection. The abdominal cavity was explored, and there was no evidence of a secondary cause of peritonitis. The peritoneal cavity was washed thoroughly. A drain was left in place.

**Figure 2:**
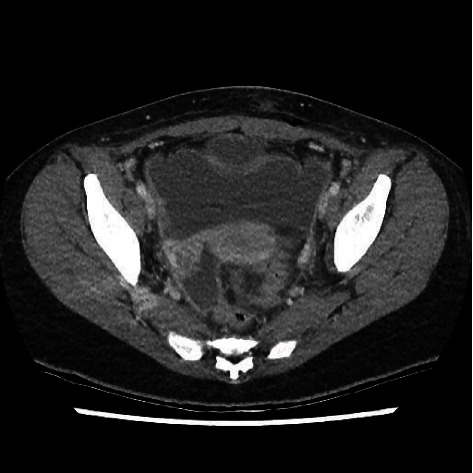
Enhanced abdominal CT scan five days after initial CT scan and first intervention showing increased amount of free pelvic fluid.

The patient was held on intravenous cefoxitin and doxycycline antibiotics. A bacterial culture from a peritoneal sample isolated GAS (isolate ID no. L00332892). Intravenous antibiotic was changed to ceftriaxone. On the third postoperative day, the abdominal drain was removed while retrieving minimal serous liquid. On the fifth postoperative day, the patient developed increased and diffuse abdominal pain. White cell count was 12,200/mm^3^, and CRP went up to 140 mg/mL (normal: 0.0–5.0 mg/mL) after reaching almost normal level. CT scan showed the presence of an increased amount of free peritoneal fluid (Figure 2). The patient was brought back to the operating room for laparoscopy and lavage. The presence of purulent fluid and false membranes on the pelvic organs were found. The abdomen was again washed thoroughly, and another drain was left in place.

The patient was kept on ceftriaxone and intravenous metronidazole was added to extend coverage. The patient gradually recovered. She was discharged nine days after the second intervention and was held on IV ceftriaxone and oral metronidazole for two more weeks. Follow-up CT scans showed pelvic collections that gradually resolved. Two months later, the patient had no residual symptoms.

Retrospectively, the patient reported having oral sex the same day after abortion. Her partner was asymptomatic, but his throat cultures were positive for the presence of GAS. He was treated with oral antibiotics.

Molecular analysis of the GAS bacterial culture from the patient (isolate ID no. L00332892) indicated an *emm* type *emm77* with a molecular profile unique from the other *39 emm77* background isolates collected in Quebec from 2016 to 2021. Unfortunately, molecular analysis of the bacterial culture of the sexual partner could not be analyzed because it was discarded.

## 3. Discussion

Peritonitis usually occurs from an abdominal source such as appendicitis, diverticulitis, or hollow viscus perforation. Primary peritonitis is an infective inflammation of the peritoneal cavity in the absence of an abdominal source [1–4]. Primary peritonitis represents less than 1% of all peritonitis [5, 6]. Considering that most primary peritonitis occurs in association with chronic hepatopathy or nephropathy, autoimmune disease, and immunosuppression, spontaneous peritonitis without predisposing factors therefore remains very unusual [1]. *Streptococcus pneumoniae* represents the first cause of spontaneous peritonitis [17] while GAS remains very rare [7].

GAS is a strictly human pathogen usually found in the skin and throat and less frequently in the rectum and the female genital tract [18]. There is considerable evidence supporting female genitalia as an entry to the peritoneal cavity for *Streptococcus* species [2, 3, 7, 9, 14, 16, 19]. Tardieu and Schmidt in 2014 [7] and Iwata and Iwase in 2017 [14] reviewed, respectively, 35 and 86 cases of GAS peritonitis (with some cases evidently retrieved in both reviews) where female to male ratios were, respectively, 4 : 1 and 7 : 1. In the review of Iwata and Iwase, 10 cases out of 55 (18%) premenopausal women had either IUCD or abortion [14]. Forty percent of cases were associated with an identified vaginal origin. Respiratory or cutaneous origins were identified in 34% of cases. This leaves 25% of cases with unknown origin [14].

The vagina was the likely bacterial entry site in the present case, possibly transmitted through oral sex with a proven GAS carrier, similar to the only other report of transmission of GAS with oral sex [16]. However, because the bacterial isolate from the sexual partner was not characterized, oral transmission cannot completely be implicated without any doubt. Ascending infection from an asymptomatic vaginal carrier is thus a possibility, along with contamination of the partner by the patient.

In the previously reported case, the patient who developed GAS peritonitis was using an IUCD [16]. Recent delivery, uterine intervention, or the presence of an IUCD are thus probably catalytic factors in case of exposure to GAS [1, 16]. The incidence of orogenital transmission of invasive GAS, while certainly very low, is likely [16] underreported and not rigorously considered a contributing factor of contamination.

Isolate L00332892 belongs to type *emm77*. The prevalence of *emm77* in Canada is low (1.77%) compared to the most common type *emm1* (12.1% in 2019) [20]. An epidemiological study on iGAS infections reported in Europe showed that distribution of *emm77* is also less predominant (2%) [21]. Interestingly, *emm77* isolates are more common among patients without focal symptoms [21] and could be also associated to genital infections [22].

GAS may progress very rapidly to severe disease [1, 2, 8–11] and incidence and severity are both increasing [12] with mortality rate that may reach as high as 45% [13]. Evolution of the disease may be so fast and dramatic [1, 2, 7–9, 11] that at least exploratory surgery is recommended by most surgeons [1, 6]. In the review of by Tardieu and Schmidt, 34 of 35 were patients taken to the operating room [7] and no deaths were reported. In our opinion, this probably represents a bias in published cases. However, one out of the three patients reported by Tardieu and Schmidt died even though all underwent surgery [7]. Afterwards, fatal cases were reported, one following vaginal delivery [9] and another one without an identified cause or origin [2]. This latter case, whose diagnosis was obtained after an abdominal puncture, passed away without undergoing surgical intervention [2].

Although stable, our patient underwent a laparoscopy to rule out a uterine perforation. At the intervention, a secondary cause could be eliminated, the peritoneal cavity could be washed, and cultures could be obtained. Although some cases may be managed without surgery [6], we recommend, like others [1, 2, 4, 7, 23], early surgery without awaiting rapid deterioration [8, 10, 11, 14] particularly that laparoscopy is now widely available as a diagnostic tool as well as a therapeutic intervention [10, 24]. In our case, besides the aggressiveness of the infection itself, initial laparoscopic lavage was possibly not sufficient.

In cases of primary peritonitis, even if diagnosis is contemplated, early surgery (except for patients with known ascites in which intervention carries no advantages) is advocated to exclude secondary peritonitis, establish a diagnosis, obtain a peritoneal fluid sample for bacterial characterization, and to drain, wash, and remove devitalized tissues from the abdominal cavity [1, 2, 4, 7]. Also, from this particular case, we explore the possible carrier status of the sexual partner.

## 4. Conclusions

Peritonitis caused by Group A *Streptococcus* is rare and could occur in puerperium after genital intervention or with the presence of intrauterine contraceptive device; however, the contamination by a sexual partner via oral sex is very unusual but should be searched for. Here, we describe a case of invasive GAS disease caused by a unique *emm77* isolate associated with another contributing gynecologic factor and possible orogenital transmission. The recovery outcome of this case indicates that early intervention should be part of an ideal case management.
